# A Case of Compression of the Spinal Cord by Dr. William L. Baldwin

**Published:** 1869-10

**Authors:** 


### A Case of Compression of the Spinal Cord by Dr. William L. Baldwin.

A young man aged about thirty-five, an architect by profession, applied on the 22d of last March (1869) for professional advice stating that five years since he was taken with a sneezing fit which produced the following results, viz : A temporary paralysis of the whole system, an excruciating pain through the chest with a great difficulty of respiration and a peculiar pain shooting through the arms, more especially the right.

This continued for the space of ten or more seconds, when some one of his friends (who were in the room with him) noticed his distress and moved him from his fixed position, thereby throwing his head forward, when the paralysis left him, and with the exception of a certrin numbness in the upper extremities, and the pain or rather soreness about the chest, he felt quite comfortable.

After a day or two all the unpleasant symptoms had passed off, the patient enjoyed good health, sleeping well, a good appetite and in fact was never better in his life.

Since which time whenever his head is thrown suddenly backward, he experiences the same sensations, as before described, and after a short time a flacid tumor about the size of a walnut makes its appearance to the right and below the *vertebra prominens*, which in connection with a stiff neck continues for several days, after which he has his usual good health until a recurrence of the attack.

He informs me that he averages two of these attacks in the course of a year, and at no time has it caused him to be absent from his work for more than two or three days. When about fifteen or sixteen years old he was troubled with fits, which from the description given me by his father, appear to have been epileptical in form.

"These, however, (to use his own language he outgrew," and has had no return of them for several years.

He never remembers to have received an injury of any kind which could in the least have any bearing upon, or cause this difficulty in the vertebral column, and with this sneezing fit before alluded to, this peculiar train of symptoms clearly indicative of compression of the cord made their first appearance. When the head is again thrown forwards, after one of these attacks, the compression ceases, and with it all the symptoms, with the exception of pain, or rather soreness and stiffness of the neck, which may be looked upon as an effect of the graver symptoms, showing conclusively, that the compression is but temporary. Upon close examination I find no displacement of the vertebræ or of any of the processes, everything appears to be in *situ*, and except when the head is thrown suddenly back, the patient is *free from* all of this trouble.

Thus far I have endeavored to give the circumstances connected with the case, its history so far as learned, the peculiar train of symptoms produced, etc. Now, however, it becomes necessary to assign some cause for this temporary compression.

Several physicians, to whom I have related the case, have advanced explanatory theories, as detatched spiculæ of bone, bony exostosis, compression and engorgement of the spinal veins, etc., but to my mind none of these reasons are either satisfactory or applicable to the case, for the compression is but temporary and does not produce any spinal irritation, therefore the substance, be it what it may, that produces this presure is soft and somewhat elastic.

To account for it, I have theorized after this manner: that there is a portion of the intervertebral cartilage partially detached and somewhat wedge shaped, and that the edge of this wedge is toward the anterior portion of the body; then, the head being thrown back (the vertebræ moving with it,) the posterior portions of their bodies are brought nearer into opposition and cause these two hard bodies to press upon this partially detatched cartilage, which, from its shape and elasticity is forced outwards and backwards, and impinges upon the cord, thus producing the presure, the result of which is temporary, for as soon as the head is carried forward, the presure which forced this cartilage out is withdrawn, and it very naturally falls back into its normal position.

This to me appears to be the most rational mode of explaining the cause of this difficulty.—*Utica Herald.*

